# Pure and mixed ordered monolayers of tetracyano-2,6-naphthoquinodimethane and hexathiapentacene on the Ag(100) surface

**DOI:** 10.3762/bjnano.10.118

**Published:** 2019-06-06

**Authors:** Robert Harbers, Timo Heepenstrick, Dmitrii F Perepichka, Moritz Sokolowski

**Affiliations:** 1 Institut für Physikalische und Theoretische Chemie, Universität Bonn, Wegelerstraße 12, 53115 Bonn, Germany; 2 Department of Chemistry, McGill University, 801 Sherbrooke Street West, Montreal, H3A 0B8, Quebec, Canada

**Keywords:** charge transfer, low-energy electron diffraction, hexathiapentacene, scanning tunneling microscopy, tetracyano**-**2,6**-**naphthoquinodimethane

## Abstract

We report on mixed ordered monolayers of the electron acceptor-type molecule tetracyano**-**2,6**-**naphthoquinodimethane (TNAP) and the electron donor-type molecule hexathiapentacene (HTPEN). This investigation was motivated by the general question which type of mixed stoichiometric structures are formed on a surface by molecules that are otherwise typically used for the synthesis of bulk charge-transfer materials. The layers were obtained by vacuum deposition on the Ag(100) surface and analyzed by low-energy electron diffraction (LEED) and scanning tunneling microscopy (STM). The formation of the mixed structure occurs spontaneously. An important motif for the structure formation is given by hydrogen bonds between the TNAP molecules. Both molecules, TNAP and HTPEN also form well-ordered monolayers on the Ag(100) surface on their own. In all structures, the molecules are adsorbed in a planar orientation on the surface. We discuss the influence of intermolecular charge transfer on the ordering in the mixed structure.

## Introduction

Ordered monolayers on surfaces that are stoichiometrically composed of two different organic molecules have attracted strong interest in recent years [1–3]. One general motivation is to find out which ordered structures are formed in relation to the compositions of the layers and the different types of substrate surfaces. This aspect leads back to a detailed understanding of both the intermolecular interactions between the different types of molecules and their respective interfacial interactions to the surfaces. Over the last years, a variety of bicomponent layers composed of π-conjugated planar molecules have been investigated [4–8]. As a result, one finds that, on metallic surfaces, indirect intermolecular charge transfer mediated by the surface plays an important role in addition to intermolecular hydrogen bonds [5].

**Figure 1:**
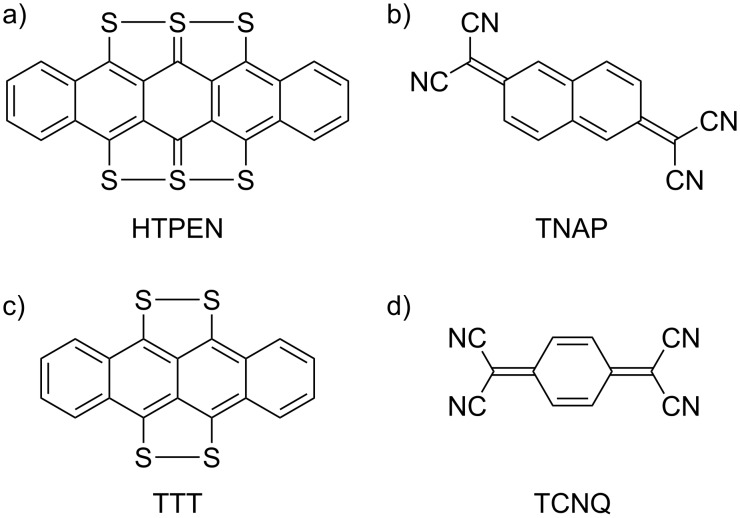
Chemical structures of (a) HTPEN, (b) TNAP, (c) TTT, and (d) TCNQ.

Particularly interesting are π-conjugated molecules with strong electron donating and accepting properties that have proved to be candidates for the formation of mixed charge-transfer (CT) crystals exhibiting interesting electric or magnetic properties [9–10]. Investigations on mixed ordered monolayers on surfaces of such molecules that are known as constituents of CT crystals thus form a specific subset. We name a few examples. On the Au(111) surface, ordered monolayers were observed for following donor/acceptor pairs: tetrathiafulvalene (TTF)/7,7,8,8-tetracyanoquinodimethane (TCNQ) [11–12], tetramethyltetrathiafulvalene (TMTTF)/TCNQ [13], α-sexithiophene (6T)/2,3,5,6-tetrafluoro-7,7,8,8-tetracyanoquinodimethane (F_4_TCNQ) [14–15], TTF/7,7,8,8-tetracyano-2,6-naphthoquinodimethane (TNAP, (Figure 1b) [16], 5,6,11,12-tetrathiotetracene (TTT, Figure 1c)/TNAP [17], and tetrabenzothianthrene (TBTA)/TNAP [17]. In addition to understand the formation of these layers from the underlying intermolecular interactions, the comparison of the intermolecular charge transfer on the metal surface in comparison with that in the respective CT crystals is of interest.

Here we report an investigation on pure and mixed layers of a pair of about equally sized planar molecules with electron donor and acceptor properties on the Ag(100) surface. The donor-type molecule was 5,6,7,12,13,14-hexathiapentacene (HTPEN, Figure 1a), which is a planar molecule with *D*_2_*_h_* symmetry [18]. Its crystal structure and field-effect mobilities were reported by Briseno and co-workers [19]. These authors [20–21] also noted that HTPEN is a one-dimensional semiconductor when it is synthesized in the form of nanowires. These nanowires exhibit a molecular structure similar to that of the bulk crystal with the primary growth direction along the [100] direction. These wires are considered to have a “great potential for use in next-generation textile and wearable electronics” [22]. HTPEN was investigated as a material for organic solar cells [23], for which purpose it was combined with lead(II) phthalocyanine and pentacene. These heterostructures exhibit a greater range of photosensitivity than pure pentacene films.

As the acceptor-type molecule we used TNAP, which is a π-extended analogue of TCNQ (Figure 1d), from which it is synthesized [24]. TCNQ and TNAP are both known to act as electron acceptors in organic CT salts [17,25]. Umbach et al. [16] found mixed layers of TNAP in combination with tetrathiafulvalene (TTF) on Au(111). TNAP is a flat planar molecule (*C*_2_*_h_*) with the ability to delocalize unpaired electrons in a charge-transfer complex [26]. We note that the crystal structure of TNAP is unknown and, as a consequence, we cannot compare the packing of TNAP on the Ag(100) surface that we will report below with that in the crystal.

HTPEN is a moderate electron donor with ca. 1 eV larger ionization potential (IP) than TTT [19]. Our own DFT calculations based on the B3-LYP functional predict the HOMO at −5.38 eV and −4.39 eV for HTPEN and TTT, respectively (the latter being in agreement with the experimental IP of 4.30–4.60 eV [27–28]). TNAP is a strong electron acceptor, but its LUMO (−4.80 eV, in good agreement with the experimental electron affinity of 4.70 [29]) is still ca. 0.5 eV above the HOMO of the HTPEN donor. (For an overview about the shapes and energies of the frontier orbitals of the respective molecules we refer to Supporting Information File 1.) Accordingly, in the gas phase one does not expect spontaneous electron transfer from HTPEN to TNAP. This in contrast to the previously studied situation for the TTT/TNAP mixed structure on the Au(111) surface [17]. However, in the case of HTPEN/TNAP on the Ag(100) surface studied here, the electron transfer (or partial charge transfer) could still occur due to the Coulomb interactions of the resulting molecular ions and due to the hybridization of molecular states with those of the metallic Ag surface. HTPEN is also interesting because of its structural similarity to TTT (Figure 1c), which has been successfully used as a donor for the preparation of mixed ordered layers with TNAP on the Au(111) surface [17]. Mixed layers containing HTPEN, instead of TTT, could further be attractive due to the expected stronger molecule–substrate bonds of the larger HTPEN molecule, which are expected to contribute to a higher thermal stability of the layers. Finally the combination of HTPEN and TNAP is interesting, because the similar size and expected footprints should allow for different tilings of the surface for a 1:1 stoichiometry.

## Experimental

The experiments were conducted in an ultra-high vacuum (UHV) chamber with a base pressure of 10^−10^ mbar. The chamber was equipped with a variable-temperature scanning tunneling microscope STM (type RHK UHV 300) from RHK Technologies and a multi-channel plate (MCP) low-energy electron-diffraction (LEED) instrument made by OCI. All LEED measurements were performed at room temperature (RT). Due to the planar screen the LEED patterns obtained with the MCP LEED do not represent undistorted projections of the reciprocal space as it is the case for a conventional hemispherical LEED optics. These intrinsic distortions were corrected using a custom-written software according to the formulas given in [30]. Nevertheless, some residual distortions still remain that typically cause a less perfect fitting of the simulated and experimental LEED patterns in the outer region of the screen. The LEED images displayed here were numerically enhanced in contrast. The simulated LEED patterns were calculated according to kinematic theory taking into account the presence of different symmetry-equivalent domains of the adsorbate.

The STM images were recorded at RT using Pt/Ir tips in constant-current mode with typical tunneling currents (*I*_t_) between 75 and 127 pA and negative bias voltages (*U*_b_), thus imaging occupied states. The displayed STM images were corrected for thermal drift distortions using the correction tool that is implemented in the SPIP software (version 4.8, Image Metrology, Denmark). The correction was done such that the unit cell of the periodic structures fitted to the unit cell determined by LEED.

All experiments were performed on the same Ag(100) single crystal, which was prepared by cycles of Ar^+^ ion sputtering at 800 eV for 30 min and annealing at 800–900 K for 60 min prior to the deposition of the molecules. HTPEN was synthesized as green microcrystalline substance by reacting pentacene with an excess of elemental sulfur in 1,2,4-trichlorobenzene solution at 210 °C, according to the published procedure [31]. The analytical data was identical to that reported in the literature [19]. The compound was additionally purified by fractional vacuum (0.1 mbar) sublimation collecting the pure HTPEN at 300 °C. TNAP is a purple substance and was bought from TCI Europe N.V. with 98% purity and used without further purification. Both substances are UHV compatible at RT. For deposition they were sublimed out of custom-built thermal evaporators onto the Ag(100) surface kept at RT. For the deposition of one monolayer (ML) of HTPEN, the respective evaporator was operated at 710 K for 3 min; in case of TNAP, at 450 K for 12 min. These temperatures are, however, not well reproducible because of variations in the thermal contact between the thermocouple and the used glass crucibles. As a result, the coverages estimated from deposition times are of limited absolute accuracy.

## Results

### HTPEN monolayer

HTPN forms two different monolayer structures on the Ag(100) surface. The first structure (termed “relaxed”) was observed as a pure phase for coverages between 0.3 and 0.5 monolayers (ML). We refer to 1 ML as the surface coverage that corresponds to a uniform and complete monolayer of the relaxed structure. However, a complete monolayer of this structure does not form, as a second structure (termed “compressed”), is observed for coverages above 0.75 ML. For intermediate coverages (0.5 to 0.75 ML) these two structures coexist. At coverages below 0.3 ML, the HTPEN molecules are mobile and form a disordered gas-like phase on the surface. This can be concluded from fluctuations in the STM current when apparently void surface regions are scanned. The occurrence of a gas-type phase is indicative of only very small or even repulsive intermolecular interactions that cannot compensate the loss in entropy related to the condensation of the HTPEN into islands.

**Figure 2:**
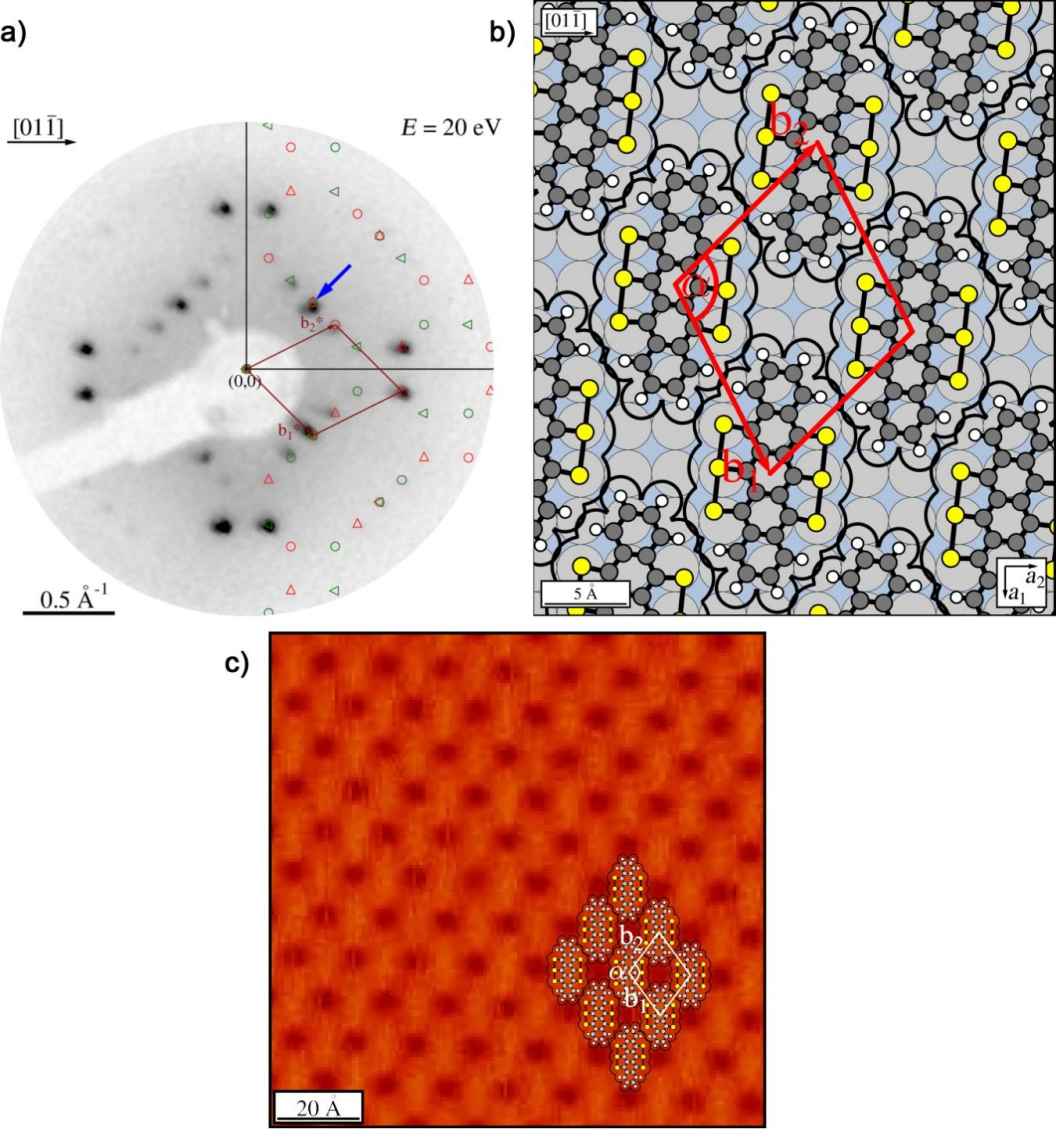
Relaxed superstructure of HTPEN on Ag(100). (a) LEED pattern at an electron energy of 20 eV. A simulated diffraction pattern is overlaid on its right half. Triangles and circles represent respective domains related by mirror symmetry; green and red spots correspond to domains rotated by 90° with respect to each other. The two overlapping spots labeled by the blue arrow are indicative for the commensurability of the superstructure. The black lines indicate the orientation of the Ag(100) substrate and aid in the identification of the symmetry of the LEED pattern. The lengths in reciprocal space are defined by k = 2π/λ, with λ denoting the electron wavelength. (b) Hard-sphere model of HTPEN on Ag(100). (c) Fourier-filtered, drift-corrected and contrast-enhanced STM image. The slow scanning direction is horizontal (*U*_b_ = −2.0 V; *I*_t_ = 75 pA). For the color coding of the atoms compare to the formulas in Figure 1.

The LEED pattern of the relaxed structure is shown in Figure 2a and yields following superstructure matrix:


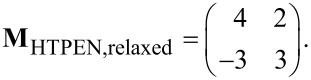


The superstructure is commensurate, which is in particular evidenced by the overlapping of the two LEED spots labeled by the blue arrow in Figure 2a. The unit cell derived from LEED measurements has a size of 150.2 Å^2^ and is large enough for containing one flat-lying HTPEN molecule, as illustrated in Figure 2b. The molecular geometry used for the hard-sphere model of HTPEN (Figure 2b) is based on the crystal structure data of HTPEN [19] and the van der Waals radii taken from [32]. Table 1 shows all geometric properties of this and all other unit cells discussed in this paper. The orientation of the molecule within the unit cell, however, could not be obtained by geometric considerations alone, because the cell shape is compatible with orienting the molecules either along its short or long diagonal. The hard-sphere model given in Figure 2b is thus based on additional information from STM images given below. The indicated adsorption of the molecule on the top-site of the Ag(100) surface in Figure 2b is not experimentally verified. This would have been possible, if we had been able to record atomically resolved STM images of clean Ag surface regions in parallel with islands of the molecules. However, this was experimentally not possible. This uncertainty concerning the adsorption sites applies also to the other hard-sphere models that we will report, e.g., that of the HTPEN/TNAP layer given below.

**Table 1:** Overview of the superstructures considered here. **M** denotes the superstructure matrix of the overlayer, *b*_1_, *b*_2_ denote the lengths of the unit cell vectors of the superstructure, α is the enclosed angle, and *A* is the area of the unit cell. The error of the integer matrix elements is smaller than 0.03.

	HTPEN		TNAP		HTPEN + TNAP
	relaxed structure	compressed structure	monolayer	multilayer	monolayer
**M**	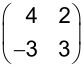	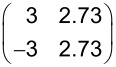	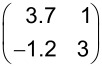	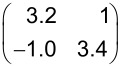	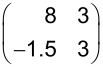

*b*_1_ [Å]	12.92	11.72	11.07	9.68	24.68
*b*_2_ [Å]	12.26	11.72	9.33	10.24	9.69
α [°]	108.4	95.4	96.7	89.0	96.0
*A* [Å^2^]	150.2	136.7	102.6	99.1	237.8

Figure 2c shows a drift-corrected and Fourier-filtered STM image taken of a large island of HTPEN molecules. The darker areas (lower tip position) between the molecules are significant. By comparison with the overlaid hard-sphere model we identify these as the voids between the molecules where a reduced density of states is present. Their arrangement reveals that the orientation of the molecules is very close to the direction of the long diagonal of the unit cell. The hard-sphere model in Figure 2b, derived from LEED, indicates an included angle of about 1.5°. The alignment of the diagonal of the unit cell and, consequently, also of the long molecule axis is not exactly in the direction of the close-packed Ag rows (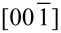 direction), which one could have expected for symmetry reasons. Instead, it is tilted by an angle of 8° with respect to this direction, as can be seen from the structure model shown in Figure 2b.

**Figure 3:**
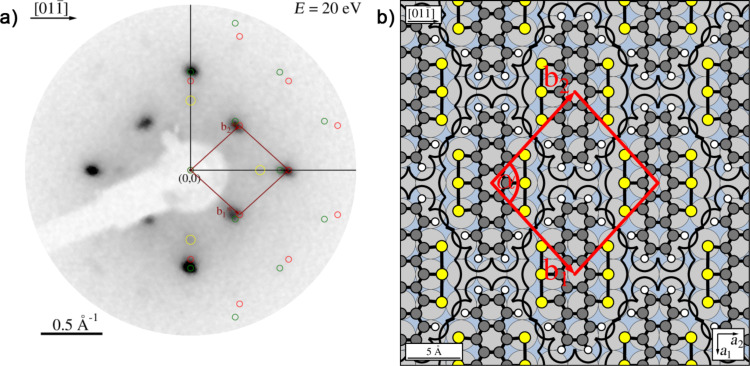
Compressed superstructure of HTPEN on Ag(100). (a) LEED pattern at an electron energy of 20 eV. A simulated diffraction pattern is overlaid. Green and red spots correspond to the two symmetry-equivalent domains rotated by 90° with respect to each other. Very faint spots marked by the yellow circles are due to multiple electron scattering. These are obtained by adding the vector of the first-order substrate spots (2.18 Å^−1^) to those of the strong second-order HTPEN spots. (b) Hard-sphere model of the compressed structure of HTPEN.

A LEED pattern of the second, compressed structure of HTPEN is shown in Figure 3a. The superstructure matrix is of the point-on-line type [33]. This is indicated by the integer numbers in the first column of the matrix:


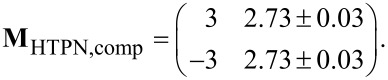


The unit cell has an area of 136.7 ± 1.5 Å^2^ and is thus by 9% smaller than the unit cell of the relaxed structure. Due to the symmetry of the diffraction pattern, only two rotational domains can be observed, but no mirror domains. The resulting real-space unit cell is of rhombic shape and is illustrated in Figure 3b. The arrangement of the molecules that we suggest here on the basis of the space requirement of the molecules is very similar to that in the relaxed structure. In both structures, the molecules are arranged in a brickwall-like structure, where the sulfur atoms along the sides of the molecules face hydrogen atoms of the terminal benzene rings of the neighboring molecules. While the long axis is tilted versus the 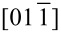 direction of the Ag(100) surface in the relaxed structure, the hard-sphere model (Figure 3b) of the compressed structure suggests that the long axis is exactly parallel to this direction in the compressed structure. As it is often the case for π-conjugated molecules adsorbed on metal surfaces, both monolayer structures are dissimilar to the molecular arrangement in the HTPEN bulk crystal [19]. In the bulk crystal, the molecules form stacks with different alternating orientations of the molecular plane [19], while here, on the Ag(100) surface, all molecules are forced into the same planar orientation by the interaction to the metal surface. Further molecules that are deposited onto the compressed first layer possibly form a disordered gas-like second layer on top of this layer. We conclude this because the STM imaging process was strongly disturbed for coverages exceeding those required for a compressed monolayer.

### TNAP layers

**Figure 4:**
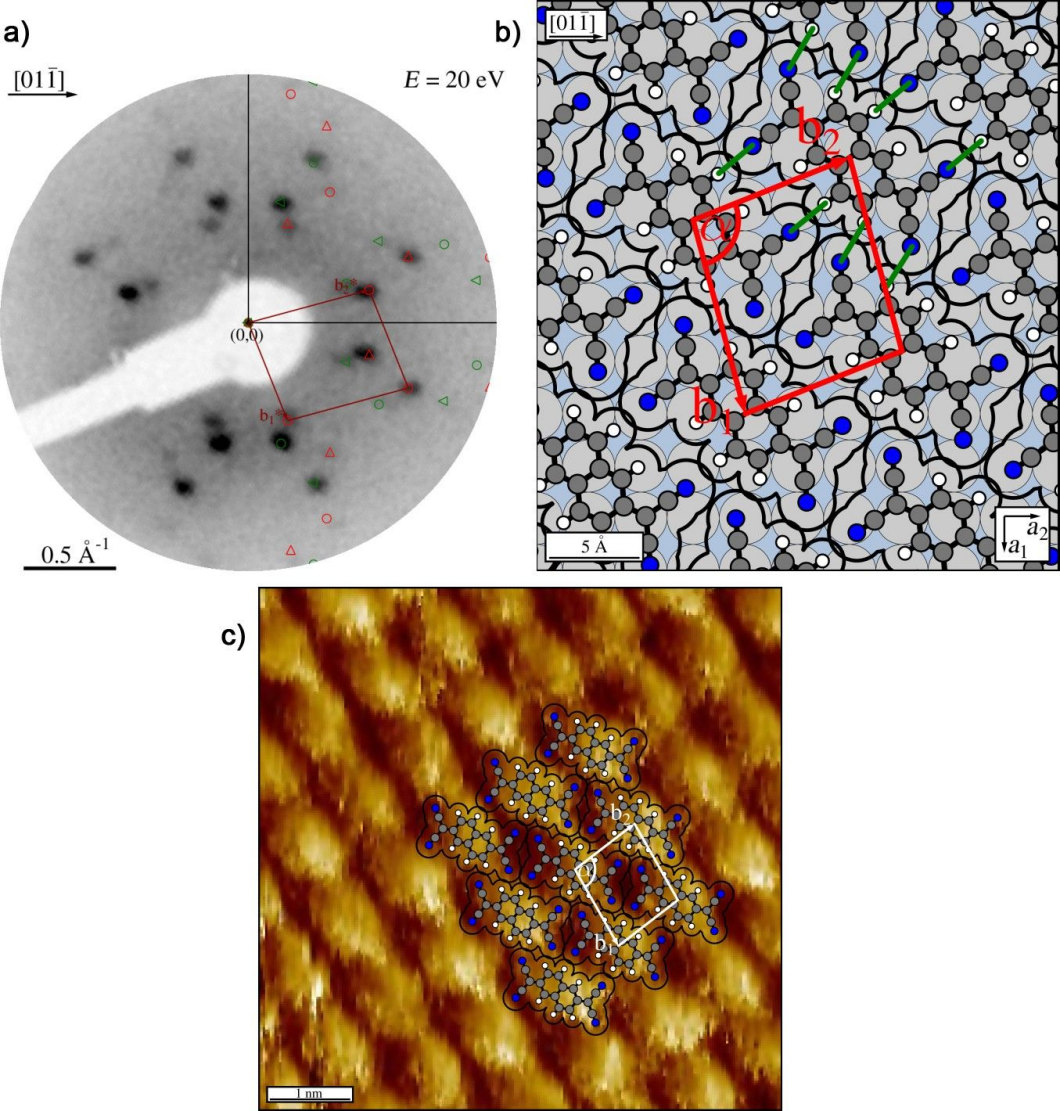
**TNAP** on Ag(100). (a) LEED pattern an electron energy of 20 eV. A simulated diffraction pattern is overlaid. Symmetry-equivalent domains are marked as in Figure 2. (b) Hard-sphere model with indicated suspected intermolecular hydrogen bonds (green lines). (c) Drift-corrected and filtered STM image. The fast scanning direction is the vertical direction (*U*_b_ = −0.81 V; *I*_t_ = 127 pA). A hard-sphere model of the structure derived from LEED has been overlaid. The non-symmetric shape of the molecule is assigned to tip effects, but not to a non-planar orientation of the molecules.

To our knowledge, the adsorption of TNAP has been studied only on Au [34] but not on Ag surfaces. The TNAP monolayers on Ag(100) were prepared under the same conditions that were used for the TNAP monolayers on Au(111) before [34]. The resulting LEED pattern is shown in Figure 4a. It can be explained by four symmetry-equivalent domains of one superstructure. The corresponding unit cell has a size of 102.6 Å^2^ ± 1.7 Å^2^. The corresponding superstructure matrix reads:


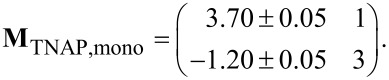


Due to the size and shape of the unit cell, there is only one possibility for the orientation of one flat-lying TNAP molecule in the unit cell, namely along the shorter diagonal of the unit cell. A hard-sphere model is shown in Figure 4b. The geometry of the TNAP molecule used for the hard-sphere model is based on Figure 1b and tabulated on interatomic distances from [35], and van der Waals radii from [32]. The structure is composed of parallel rows of molecules aligned along their long axes. The major intermolecular interactions are given by hydrogen bonds between the four cyano groups at the corners and hydrogen atoms on the long sides of the molecule. In contrast to the relaxed HTPEN structure, the TNAP superstructure is not commensurate, but of the point-on-line type [33,36]. Quite generally, the point-on-line registry of a monolayer indicates a smaller corrugation of the bonding potential at the organic/metal interface in relation to intermolecular interactions compared to a commensurate structure. Briefly speaking, this is due to the fact that the lattice of the adsorbate and the substrate have less common Fourier components and that the energy gain related to the interfacial interactions is thus smaller [33].

STM images (Figure 4c) show the formation of well-ordered and defect-free domains, with a structure consistent with the LEED pattern. Domains on flat terraces exhibit a lateral extension of more than 100 nm, but they do not overgrow Ag steps. The molecules appear to lie flat on the surface with a higher apparent height of the center of the TNAP molecules, possibly related to the comparably electron-rich aromatic core of the molecule. At lower coverages, the nucleation of small TNAP islands (below 50 nm in diameter) at step edges and on terraces was observed. Hardly any mobile molecules could be found between the islands, as seen from a stable and smooth tunneling current without spikes due to molecules diffusing in and out of the tunnel gap. This indicates the presence of strong intermolecular attractions that lead to the formation of two-dimensional islands surrounded by a two-dimensional gas phase of molecules with only low density.

**Figure 5:**
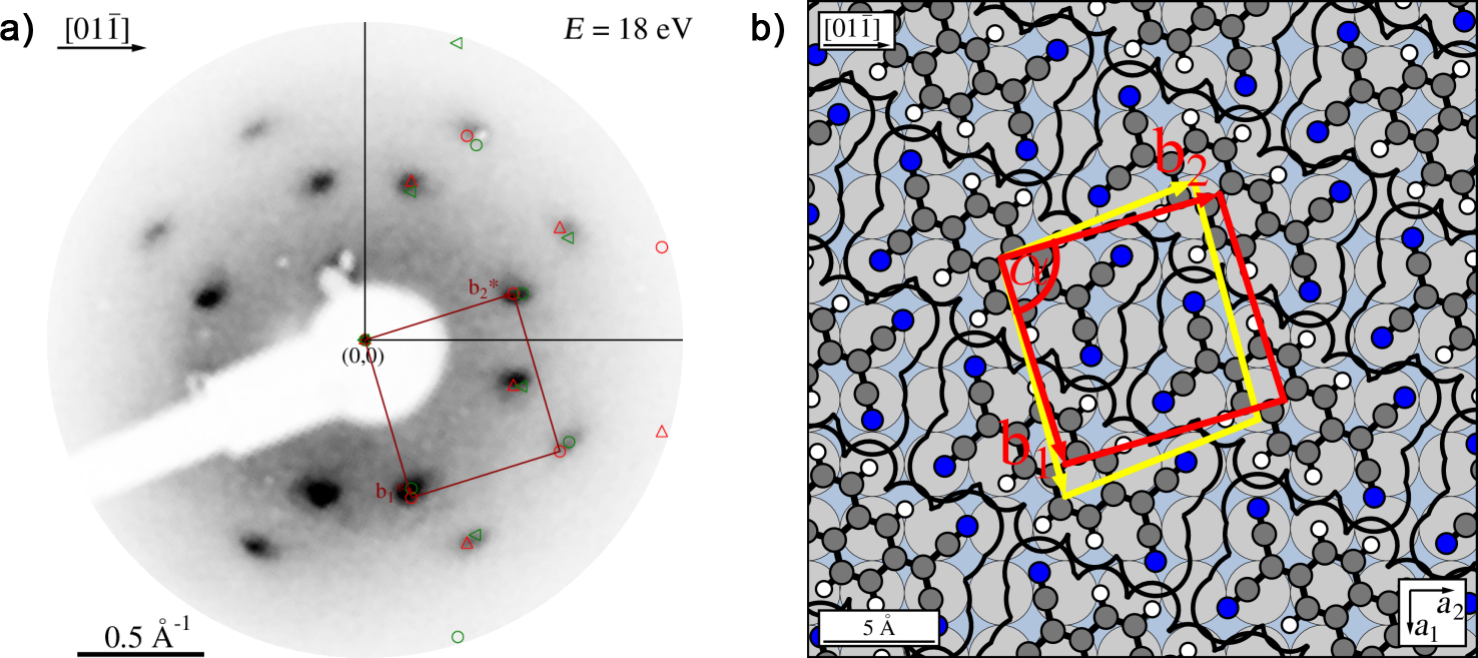
Multilayers of TNAP on Ag(100). (a) LEED pattern recorded at an electron energy of 18 eV. A simulated diffraction pattern is overlaid. Symmetry-equivalent domains are marked as in Figure 2. (b) Hard-sphere model of the TNAP multilayer (red unit cell); in addition, the unit cell of the TNAP monolayer (yellow) has been superimposed.

After depositing an amount of molecules considerably larger than that needed for a complete monolayer of the described structure, the LEED pattern changed (Figure 5a). The corresponding unit cell (99.1 ± 7 Å^2^) is by 3.5% smaller than the unit cell of the monolayer. The substrate LEED spots could not be detected any more. We interpret this by the formation of ordered multilayers of TNAP. Diffraction spots of these multilayers could only be observed at lower electron energies, up to 40 eV; at higher energies, no spots at all were distinguishable against the background.

The hard-sphere model of the unit cell (Figure 5b) is based on the assumption of flat-lying molecules. It indicates a ca. 0.5 Å overlap of the van der Waals radii of the peripheral atoms. We consider this as meaningful, although there is some uncertainty about the exact size of the footprint of the molecule on the Ag surface, because we drew the molecule using standard tabulated bonding lengths [35], and because intermolecular bond lengths may be modified due to the interactions with the substrate [37]. We thus speculate that the lateral packing in the multilayers might involve a small out-of-plane rotation of the TNAP molecules around their long axes, leading to a small tilt of the molecules towards one of their long sides. This yields a roof tile-like arrangement and a reduction of the molecular effective footprints, which reduces the intermolecular overlap.

The TNAP multilayers exhibit a superstructure matrix, which is not of the point-on-line type anymore, but indicates an incommensurate structure:


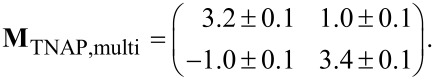


In Figure 5b both unit cells are shown, the one of the monolayer in yellow and that of the multilayers in red color. From the comparison of the hard-sphere models of both structures (cf. Figure 4b and Figure 5b), we find that the relative arrangement of the rows of TNAP molecules with respect to each other differs for both structures. However, from the positions of the peripheral hydrogen atoms with respect to the nitrogen atoms it appears that very similar hydrogen bonds as in the monolayer are important for the intermolecular interactions in the multilayers, too.

### Mixed HTPEN/TNAP monolayer

**Figure 6:**
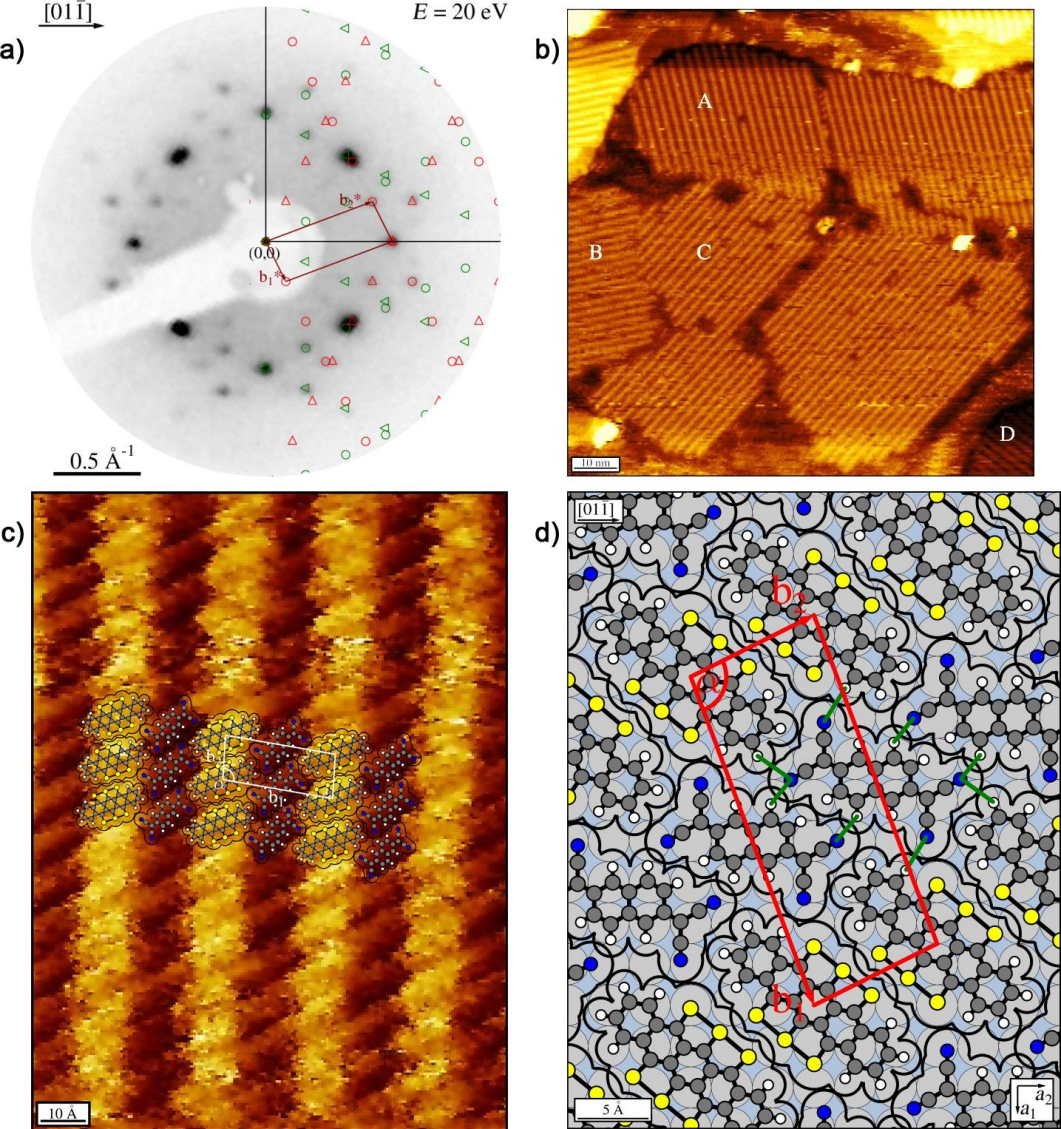
Mixed structure formed by HTPEN and TNAP on Ag(100). (a) LEED pattern recorded at an electron energy of 20 eV. A simulated diffraction pattern is overlaid. Symmetry-equivalent domains are marked as in Figure 2. (b) STM image showing different islands. Here, all four possible domains with different orientations can be observed at the same time. They are marked by A–D. The fast scanning direction is in horizontal orientation (*U*_b_ = −1.51 V; *I*_t_ = 82 pA). This STM image has been selected to demonstrate the presence of the different symmetry-equivalent domains. The corresonding islands are smaller than usual. (c) STM image. The fast scanning direction is horizontal (*U*_b_ = −0.80 V; *I*_t_ = 80 pA). A hard-sphere model of the structure derived from LEED has been superimposed. (d) Hard-sphere model with indicated unit cell and suspected hydrogen bonds (green lines) to nitrogen atoms of the TNAP.

When about equal amounts of both molecules were deposited, we immediately observed a LEED pattern that differs from the two LEED patterns of the pure layers (Figure 6a). No additional annealing step was required, and the same LEED pattern was seen independently from the sequence of the deposition of the two types of molecules. Thus, a new mixed ordered structure of the two molecules forms spontaneously. Residual LEED spots related to the pure phases were not found, which indicates that a large fraction of molecules participates in the newly formed mixed phase. It must be thus thermodynamically preferred with respect to the pure phases, causing exclusively the formation of this structure. The LEED pattern yields a superstructure matrix that is commensurate with the substrate surface in second order:


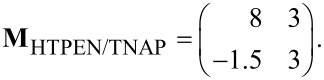


The unit cell exhibits an elongated shape and contains one flat-lying molecule of each type. This can be concluded from the unit-cell size of 237.8 Å^2^, which amounts to about 99% of the sum of the areas of the unit cells of the relaxed HTPEN monolayer phase and the TNAP monolayer phase (136.7 Å^2^ (HTPEN) + 102.6 Å^2^ (TNAP) = 239.3 Å).

The structure consists of alternating rows formed by HTPEN and TNAP molecules. This arrangement is illustrated by the hard-sphere model in Figure 6d. The rows run along the short unit cell vector **b**_2_, and form an angle of 26.6° with the 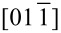 direction of the surface. On the basis of the symmetry of the Ag(100) surface (space group *P*4*mm*) and the symmetry of the molecules (HTPEN: *D*_2_*_h_*, TNAP: *C*_2_*_h_*) four symmetry-equivalent domains with four different orientations of the rows can be expected. All of these possible orientations could be observed in STM images. Figure 6b displays an example. The four symmetry-equivalent domains are marked by A to D. The molecular rows are well visible. The domains exhibit local defects, mostly vacancies, where single molecules are missing. For this structure, STM images of good quality with a low level of noise were obtained easily. We interpret this by the fact that there are no, or only very few, mobile molecules on the surface which could otherwise cause variations in the tunnel current by moving in and out of the tunnel gap.

A significant contrast in the apparent height between the rows of the two types of molecules is found (Figure 6c). We suggest that this contrast indicates different energies of the highest occupied molecular orbitals with respect to the Fermi level. Geometric height differences may also play a role, but because both molecules lie flat we consider them as less relevant. In principle, we can expect charge transfer from the HOMO of the donor-type molecule, HTPEN, towards the LUMO of the acceptor-type molecule, TNAP. The STM images, shown in Figure 6b and Figure 6c, were taken at negative bias voltages (−1.5 V and −0.8 V, respectively), and thus image orbitals below the Fermi energy *E*_F_. On the Ag surface, the charge transfer is mediated by the electrons of the substrate, and, consequently both interacting orbitals exhibit at least some density of states at the Fermi edge. For the interpretation of the tunnel contrast and the assignment of the molecules, we use information from the mixed phase of TTT and TNAP on the Au(111) surface [17], because our observed mixed structure bears great similarities to that of mixed layers of TTT and TNAP found on Au(111) [34]. In both systems, alternating parallel rows of one type of molecule are formed, yielding a one-to-one stoichiometry of acceptor and donor molecules. In the mixed structure of TTT and TNAP on Au(111) the brighter molecules could be assigned to the donor type TTT molecules, while the TNAP molecules appeared darker. Hence, we propose that for HTPEN/TNAP the high density of states leading to the bright rows in the STM images at negative bias voltages is also related to the donor type molecule and thus the partially depopulated HUMO of the HTPEN.

The unit cell of the mixed phase is commensurate in second order. The commensurability indicates that the surface is relevant for the lateral ordering. The commensurability in second order implies that every second HTPEN and TNAP molecule sits exactly on the same adsorption sites on the Ag surface. Notably, the azimuthal orientation of the HTPEN molecules with respect to the substrate in the mixed HTPEN/TNAP phase differs significantly from that in the pure HTPEN phase (cf. Figure 2b and Figure 6d), whereas the orientation of the TNAP molecule is rather similar in both structures. Because the pure HTPEN phase is commensurate, while the pure TNAP phase is of the point-on-line type, we propose that the stronger adsorbate/substrate interaction of the HTPEN is responsible for the commensurability of second order of the mixed structure.

In the mixed structure, the orientation of the TNAP molecules with respect to each other is almost the same as that in the pure layers and exactly the same as that observed by Fiedler et al. [17] for TNAP on Au(111). Every nitrogen atom is surrounded by several hydrogen atoms from neighboring TNAP and HTPEN molecules. The latter are azimuthally oriented in such a way that the hydrogen atoms at the end of the HTPEN molecule directly point at the nitrogen atoms of the nearest TNAP molecule. Within the TNAP rows, the molecules are arranged in such a way that the nitrogen atoms on neighboring molecules are as far apart from each other as possible. This arrangement suggests the presence of strong hydrogen bonds between nitrogen atoms of the TNAP and hydrogen atoms of both TNAP and HTPEN molecules. In this mixed structure, the smallest distance between sulfur atoms of neighboring HTPEN molecules is significantly smaller (30%) than that between neighboring HTPEN molecules in the pure HTPEN phases (cf. Figure 2b and Figure 3b). This is possibly supported by the partial charge transfer from HTPEN to TNAP. A reduction of the electron density at the sulfur atoms allows neighboring HTPEN molecules to arrange closer to each other than in the pure HTPEN layer.

Overall a very closely packed and stable monolayer of HTPEN and TNAP is formed, which is indicative for strong attractive intermolecular interactions. Nevertheless, the commensurability of the structure in second order also suggests site-specific interactions between the molecules and the Ag(100) surface.

## Discussion

Both molecules (HTPEN and TNAP) form ordered monolayers on Ag(100) in the form of pure phases and also as a binary mixed phase. All monolayers are thermodynamically stable at room temperature, which means in particular that no de-wetting from the Ag(100) surface by formation of small three-dimensional clusters occurred over time.

HTPEN forms two different structures on Ag(100) depending on the coverage. The predominantly observed phase has a slightly larger unit cell (ca. 10%) than the second phase with a more compressed structure. The intermolecular interactions of the flat-lying HTPEN molecules on the Ag(100) surface appear to be very weak or possibly even repulsive. This is suggested by the fact that ordered domains are only formed at coverages exceeding a critical coverage of about one third of that of a complete monolayer (at room temperature). At lower coverages, only a disordered and gas-like phase was observed, similarly to the situations found, e.g., for TTT on Au(111) [34], tetracene on Ag(111) [38–39], hexa-*peri*-hexabenzocoronene on Au(111) [40], and Sn phthalocyanine on Ag(111) [41]. This behavior differs from that of other organic molecules that exhibit attractive intermolecular interactions, e.g., PTCDA (perylenetetracarboxylic dianhydride) on Ag(100) [42], and for which the formation of ordered islands is observed already at very small coverages. In the present case of presumably weak or repulsive intermolecular interactions, the ordering at higher coverages is driven by the attractive interactions between the HTPEN molecules and the Ag substrate. These support ordered structures because disordered structures of chain-like long molecules do not lead to high coverages (due to empty regions of surface between the molecules) and thus provide only a limited adsorption energy per area. We propose that the repulsive intermolecular interactions that we discussed above are related to negative partial charges on the electron-rich sulfur atoms at the periphery of the HTPEN molecule. The repulsion between the equally oriented dipoles related to the molecule/surface charge transfer may also play a role but is supposed to be less important here. Notably, in the three-dimensional bulk structure of HTPEN, attractive electrostatic bonds between the central sulfur atoms and the outer sulfur atoms were deduced from small S–S distances (3.37–3.41 Å) between molecular stacks [19]. However, these interactions are less relevant on the surface, because the confined geometry of the two-dimensional packing on the surface does not allow for the same intermolecular arrangement as in the bulk.

Due to a combination of only weak attractive or even repulsive intermolecular forces between the molecules and a relevant corrugation of the adsorption potential on the Ag(100) surface, the HTPEN molecules form ordered islands with a commensurate structure. At coverages above 0.5 ML this commensurate structure transforms into a compressed structure of the point-on-line type with a by 9% smaller unit cell. Presumably, the related gain in the adsorption energy per area overcompensates the loss in interfacial energy due to the lifting of the registry of the structure with the substrate (commensurate/point-on-line) and the increasing repulsive lateral interactions due to the smaller intermolecular distances.

TNAP also forms a well-ordered monolayer on Ag(100). The molecules are closely packed and the molecular arrangement is dominated by hydrogen bonds. These lead to attractive intermolecular interactions that cause the formation of small islands at low coverages, different to the situation seen for HTPEN. The corrugation of the interaction potential between TNAP and the Ag(100) surface atoms seems to be weaker, compared to that of HTPEN, or it is overruled to some degree by the attractive intermolecular interactions, because the structure is of the point-on-line type and not commensurate.

For TNAP of a thickness above one monolayer, we find a lateral compression of the area of the unit cell by 3.5%. Considering the intermolecular overlap deduced from a hard-sphere model, we speculated that a small out-of-plane tilting of the TNAP molecules in the monolayer (and the second layer) occurs, when the TNAP monolayer is overgrown by a second layer. This is typical for some planar organic π-conjugated molecules and related to molecule/surface interactions that favor a planar orientation for a single monolayer, but which are not strong enough to maintain this orientation, when the molecules are additionally subject to attractive interactions at the interface between the monolayer and a second molecular layer. Such a behavior was found earlier, e.g., for tetracene on Ag(111) [43].

The subsequent deposition of HTPEN and TNAP molecules yielded a stable binary monolayer. This layer is well-ordered and thermodynamically very stable, because only very few structural defects were observed. The formation occurs spontaneously and is independent of the sequence of the deposition. The strong corrugation of the bonding potential of HTPEN on the Ag surface is possibly, the reason for the commensurability of this structure. We deduce that the repulsive interactions between the HTPEN molecules, present in the pure HTPEN layer, are absent in the mixed HTPEN/TNAP layer, because we observe formation of ordered islands. We suppose that a substrate-mediated intermolecular charge transfer from HTPEN to TNAP molecules leads to attractive coulombic interactions between the HTPEN and TNAP molecules and thus leads to the formation of the densely packed structure consisting of alternating rows of HTPEN and TNAP molecules. The structure is very similar to that formed by TTT and TNAP on the Au(111) surface [17]. We propose that the charge transfer from the TTT and the HTPEN to the TNAP is thus also similar. For TTT and TNAP the details were derived from density functional theory calculations in ref. [17].

In addition, the mixed HTPEN/TNAP structure is stabilized by hydrogen bonds between neighboring TNAP molecules and between TNAP and HTPEN molecules. Between neighboring TNAP molecules there are two complementary hydrogen bonds between H and N atoms (Figure 6d). In addition to these two bonds, the structure model shows a pair of two opposing hydrogen atoms between these two bonds. This motif of two intermolecular hydrogen bonds with an intermediate pair of two opposing hydrogen atoms was also found in the pure TNAP monolayer and in the mixed structures of TNAP/TTF [16], TNAP/TTT [17], and TNAP/TBTA [17]. As far as we know, HTPEN is the donor-type molecule with the largest number of sulfur atoms for which mixed CT layers on surfaces have been observed so far. The preparation of mixed bulk crystals of HTPEN and TNAP, or other electron acceptor-type molecules, appears attractive.

## Conclusion

We prepared pure and mixed ordered monolayer structures of HTPEN and TNAP on the Ag(100) surface. The unit cells contain one molecule (pure phases) or two different molecules (mixed phase). From the structure models of the related unit cells it is deduced that the molecules lie flat on the surface. The corrugation of the Ag(100) surface binding potential appears to be important for both HTPEN and TNAP, which is indicated by the formation of commensurate or point-on-line structures. For the mixed structure of HTPEN and TNAP we find a highly ordered arrangement of the molecules in alternating parallel rows formed by the two types of molecules. The structure is stabilized by hydrogen bonds within and between the rows.

## Supporting Information

File 1 Frontier orbitals of molecules under study.

## References

[1] Bouju X, Mattioli C, Franc G, Pujol A, Gourdon A (2017). Chem Rev.

[2] Kumar A, Banerjee K, Liljeroth P (2017). Nanotechnology.

[3] Goronzy D P, Ebrahimi M, Rosei F, Arramel, Fang Y, De Feyter S, Tait S L, Wang C, Beton P H, Wee A T S (2018). ACS Nano.

[4] Stadtmüller B, Lüftner D, Willenbockel M, Reinisch E M, Sueyoshi T, Koller G, Soubatch S, Ramsey M G, Puschnig P, Tautz F S (2014). Nat Commun.

[5] Goiri E, Matena M, El-Sayed A, Lobo-Checa J, Borghetti P, Rogero C, Detlefs B, Duvernay J, Ortega J E, de Oteyza D G (2014). Phys Rev Lett.

[6] Cottin M C, Schaffert J, Sonntag A, Karacuban H, Möller R, Bobisch C A (2012). Appl Surf Sci.

[7] de Oteyza D G, Garcia-Lastra J M, Corso M, Doyle B P, Floreano L, Morgante A, Wakayama Y, Rubio A, Ortega J E (2009). Adv Funct Mater.

[8] de Oteyza D G, Silanes I, Ruiz-Osés M, Barrena E, Doyle B P, Arnau A, Dosch H, Wakayama Y, Ortega J E (2009). Adv Funct Mater.

[9] Dressel M (2007). Naturwissenschaften.

[10] Fraxedas J (2002). Adv Mater (Weinheim, Ger).

[11] Fernández-Torrente I, Franke K J, Pascual J I (2008). Phys Rev Lett.

[12] Gonzalez-Lakunza N, Fernández-Torrente I, Franke K J, Lorente N, Arnau A, Pascual J I (2008). Phys Rev Lett.

[13] Fernández-Torrente I, Kreikemeyer-Lorenzo D, Stróżecka A, Franke K J, Pascual J I (2012). Phys Rev Lett.

[14] Jäckel F, Perera U G E, Iancu V, Braun K-F, Koch N, Rabe J P, Hla S-W (2008). Phys Rev Lett.

[15] Braun K-F, Hla S W (2008). J Chem Phys.

[16] Umbach T R, Fernandez-Torrente I, Ladenthin J N, Pascual J I, Franke K J (2012). J Phys: Condens Matter.

[17] Fiedler B, Reckien W, Bredow T, Beck J, Sokolowski M (2014). J Phys Chem C.

[18] Gorishnyi M P (2006). Ukr Fiz Zh (1991-2007).

[19] Briseno A L, Miao Q, Ling M-M, Reese C, Meng H, Bao Z, Wudl F (2006). J Am Chem Soc.

[20] Briseno A L, Mannsfeld S C B, Reese C, Hancock J M, Xiong Y, Jenekhe S A, Bao Z, Xia Y (2007). Nano Lett.

[21] Briseno A L, Mannsfeld S C B, Lu X, Xiong Y, Jenekhe S A, Bao Z, Xia Y (2007). Nano Lett.

[22] Min S-Y, Kim T-S, Lee Y, Cho H, Xu W, Lee T-W (2015). Small.

[23] Lutsyk* P, Vertsimakha Y (2008). Mol Cryst Liq Cryst.

[24] Johnson G R, Miles M G, Wilson J D (1976). Mol Cryst Liq Cryst (1969-1991).

[25] Sandman D J, Garito A F (1974). J Org Chem.

[26] Garito A F, Heeger A J (1974). Acc Chem Res.

[27] Ishii H, Sugiyama K, Yoshimura D, Ito E, Ouchi Y, Seki K (1998). IEEE J Sel Top Quantum Electron.

[28] Inokuchi H, Kochi M, Harada Y (1967). Bull Chem Soc Jpn.

[29] Kanai K, Akaike K, Koyasu K, Sakai K, Nishi T, Kamizuru Y, Nishi T, Ouchi Y, Seki K (2009). Appl Phys A: Mater Sci Process.

[30] Mom R V, Hahn C, Jacobse L, Juurlink L B F (2013). Surf Sci.

[31] Goodings E P, Mitchard D A, Owen G (1972). J Chem Soc, Perkin Trans 1.

[32] Bondi A (1964). J Phys Chem.

[33] Hooks D E, Fritz T, Ward M D (2001). Adv Mater (Weinheim, Ger).

[34] Fiedler B, Rojo-Wiechel E, Klassen J, Simon J, Beck J, Sokolowski M (2012). Surf Sci.

[R35] 35We used bond lenghs for TNAP as follows: C–C: 150 pm; C=C 134 pm, C–H: 109 pm, and C–N: 120 pm.

[36] Mannsfeld S C B, Fritz T (2004). Phys Rev B.

[37] Meyerheim H L, Gloege T, Sokolowski M, Umbach E, Bäuerle P (2000). Europhys Lett.

[38] Langner A, Hauschild A, Fahrenholz S, Sokolowski M (2005). Surf Sci.

[39] Soubatch S, Kröger I, Kumpf C, Tautz F S (2011). Phys Rev B.

[40] Wagner C, Kasemann D, Golnik C, Forker R, Esslinger M, Müllen K, Fritz T (2010). Phys Rev B.

[41] Stadler C, Hansen S, Kröger I, Kumpf C, Umbach E (2009). Nat Phys.

[42] Ikonomov J, Schmitz C H, Sokolowski M (2010). Phys Rev B.

[43] Sueyoshi T, Willenbockel M, Naboka M, Nefedov A, Soubatch S, Wöll C, Tautz F S (2013). J Phys Chem C.

