# Supplementary material for: Pure and mixed ordered monolayers of tetracyano-2,6-naphthoquinodimethane and hexathiapentacene on the Ag(100) surface
Source: Beilstein J Nanotechnol. 2019 Jun 6;10:1188–99. doi: 10.3762/bjnano.10.118 (PMC6604726; doi:10.3762/bjnano.10.118)
Supplement: File 1 — Frontier orbitals of molecules under study. [file Beilstein_J_Nanotechnol-10-1188-s001.pdf]

BEILSTEIN JOURNAL OF NANOTECHNOLOGY

Supporting Information

for

Pure and mixed ordered monolayers of tetracyano-2,6-naphthoquinodimethane and hexathiapentacene on the Ag(100) surface

Robert Harbers, Timo Heepenstrick, Dmitrii F. Perepichka and Moritz Sokolowski

Beilstein J. Nanotechnol. **2019,** *10,* 1188–1199. doi:10.3762/bjnano.10.118

Frontier orbitals of molecules under study

License and Terms: This is a supporting information file under the terms of the Creative Commons Attribution License (http://creativecommons.org/licenses/by/4.0). Please note that the reuse, redistribution and reproduction in particular requires that the authors and source are credited.
The license is subject to the *Beilstein Journal of Nanotechnology* terms and conditions: (https://www.beilstein-journals.org/bjnano)

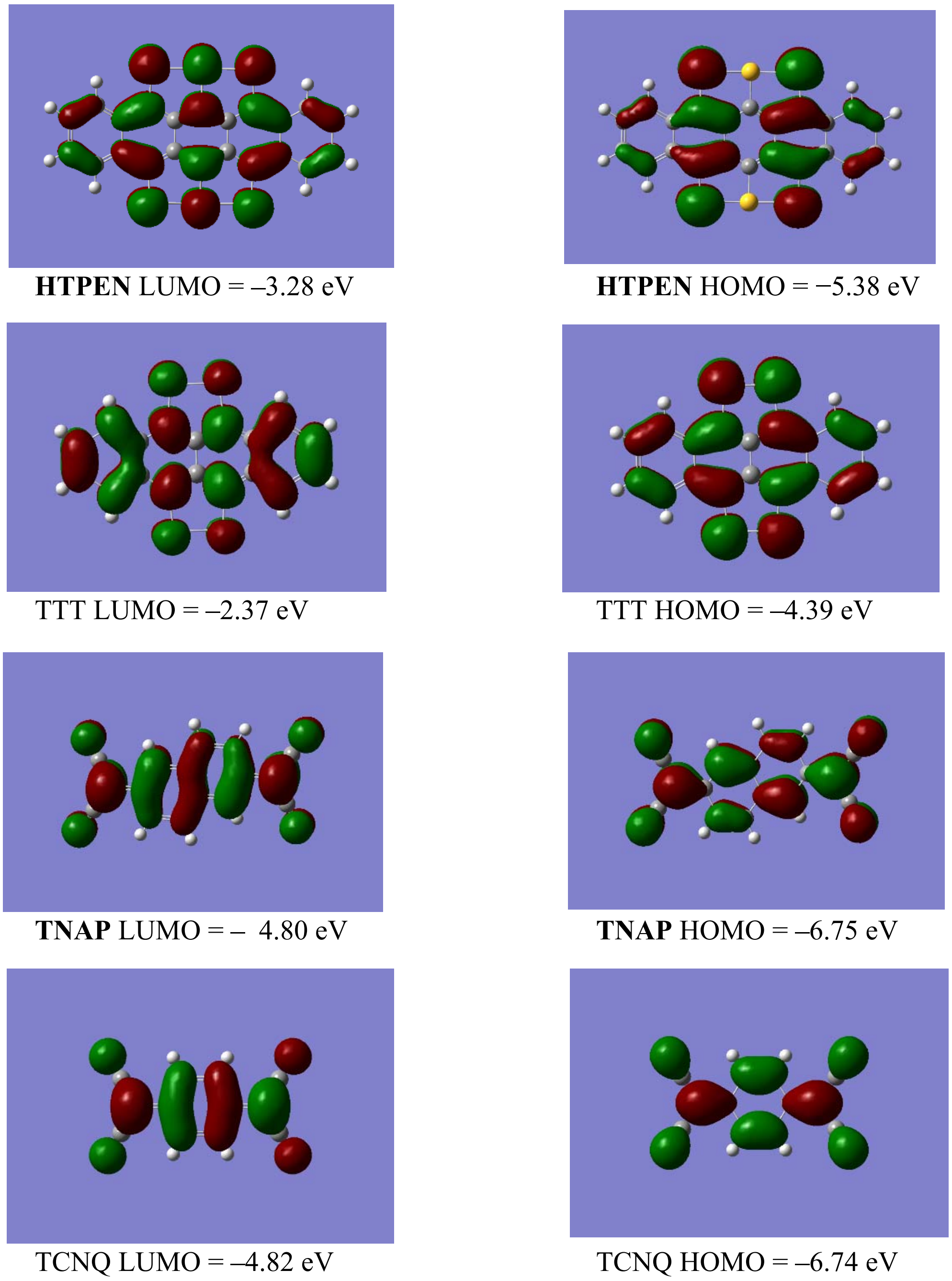

Figure S1: Frontier orbitals (LUMO and HOMO) and orbital energies of 5,6,7,12,13,14-hexathiapentacene (**HTPEN**), 5,6,11,12-tetrathiotetracene (TTT), 7,7,8,8-tetracyano-2,6-naphthoquinodimethane (**TNAP**) and 7,7,8,8-tetracyanoquinodimethane (TCNQ).
